# A TIL-Type Serine Protease Inhibitor Involved in Humoral Immune Response of Asian Corn Borer *Ostrinia furnaculis*

**DOI:** 10.3389/fimmu.2022.900129

**Published:** 2022-05-16

**Authors:** Ruobing Guan, Shaoru Hu, Xiang Li, Shiheng An, Xuexia Miao, Haichao Li

**Affiliations:** ^1^ Henan International Laboratory for Green Pest Control, College of Plant Protection, Henan Agricultural University, Zhengzhou, China; ^2^ Key Laboratory of Insect Developmental and Evolutionary Biology, Chinese Academy of Sciences (CAS) Center for Excellence in Molecular Plant Sciences, Shanghai Institute of Plant Physiology and Ecology, Chinese Academy of Sciences (CAS), Shanghai, China; ^3^ Biobank of Ninth People’s Hospital, Shanghai Jiao Tong University School of Medicine, Shanghai, China

**Keywords:** *Ostrinia furnacalis*, serine protease, proteinase inhibitor, immune, melanization, antimicrobial peptides

## Abstract

To elucidate the application value of insect endogenous protease and its inhibitor genes in pest control, we analyzed in detail the transcriptome sequence of the Asian corn borer, *Ostrinia furnacalis*. We obtained 12 protease genes and 11 protease inhibitor genes, and comprehensively analyzed of their spatiotemporal expression by qRT-PCR. In which, a previous unstudied serine protease inhibitor gene attracted our attention. It belongs to the canonical serine proteinase inhibitor family, a trypsin inhibitor-like cysteine-rich domain (TIL)-type protease inhibitor, but its TIL domain lacks two cysteine residues, and it was named as *ACB-TIL*. Its expression level is relatively very low in the absence of pathogen stimulation, and can be up-regulated expression induced by Gram-negative bacteria (*Escherichia coli*), virus (BmNPV), and dsRNA (dsEGFP), but cannot be induced by fungus spores (*Metarrhizium anisopliae*). Prokaryotic expressed ACB-TIL protein can significantly inhibit the melanization *in vitro*. Injecting this protein into insect body can inhibit the production of antimicrobial peptides of attacin, lebocin and gloverin. Inhibition of *ACB-TIL* by RNAi can cause the responses of other immune-, protease- and inhibitor-related genes. ACB-TIL is primarily involved in Asian corn borer humoral immunity in responses to Gram-negative bacteria and viruses. This gene can be a potential target for pest control since this will mainly affect insect immune response.

## Introduction

Insects rely on their innate immune system to defend exogenous pathogens (1, 2). The innate immune is a very sensitive system, which is completed by cellular and humoral immunity. Cellular immunity is mainly through phagocytosis of invading microorganisms by plasma cells, humoral immunity mainly includes the melanization response and the release of antimicrobial peptides regulated by Toll and IMD pathways (3, 4). Studies have shown that a series of serine proteases and inhibitors are involved in the insect humoral immune process, and maintain normal growth and development process of insects (5–8).

Serine protease inhibitors (SPIs) are ubiquitous in all species and can be classified as serpins, canonical inhibitors, and non-canonical inhibitors according to their functions. Among them, canonical inhibitors include trypsin inhibitor-like cysteine-rich domain (TIL), Kunitz, Kasal or Bowman-Birk according to their structural motifs (9, 10). SPIs can inhibit the activity of serine proteases by combining with them to form stable covalent complexes, thereby regulating insect humoral immunity, such as participation in the melanization reaction and resistance to fungal infections (5, 8, 11).

TIL-type protease inhibitors have antimicrobial activity. In the silk of silkworm, TIL protease inhibitors provide effective protection to silkworm pupa by inhibiting extracellular proteases secreted by pathogens (12, 13). In *Cotesia vestalis*, the CvT-TIL protein inhibits the activation of host prophenoloxidase and regulates host humoral immune mechanism (14). However, to date, we know relatively little about TIL-type protease inhibitors, only few TIL domain protein were reported (15). The Asian corn borer, *Ostrinia furnacalis* (Guenée) (Lepidoptera: Crambidae), is an important insect pest in Asia, causing serious damage to corn, sorghum, and millet (16, 17). Yield losses caused by Asian corn borer are more than 10%, in areas where the damage is severe, there may even result in a corn crop failure (18). There have some studies on the immune-related functions of Asian corn borer, however, the mechanism of melanization and antibacterial inhibition is poorly understood (19). In this study, we performed a comprehensive analysis of the spatiotemporal expression patterns of 12 protease genes and 11 protease inhibitor genes of the Asian corn borer, and cloned a TIL-type serine protease inhibitor gene and analyzed its role in melanization and antimicrobial peptide production. Our study provides some information about serine proteases and protease inhibitors, and provides a basis for further understanding of the molecular mechanism of natural immunity in Asian corn borer.

## Materials and methods

### Insect Culture

The *O. furnacalis* eggs were originally obtained from fields in Shanghai, China, and reared in the laboratory for more than 10 generations at 25 ± 1°C and 75% relative humidity on a 14/10 h light/dark cycle. The larvae were fed on a modified artificial diet (120 g maize granules, 32 g maize flour, 120 g soybean flour, 4 g vitamin C, 12 g agar, 72 g yeast powder, 4 g sorbic acid, 60 g glucose, 1.6 mL formaldehyde, and 1000 mL water). Moths were fed on 10% (vol/vol) honey solution.

### Sampling of Different Tissues and Developmental Stages of Asian Corn Borer

In order to analyze the expression profile of serine protease and inhibitor genes in different tissues, we selected the identical fifth instar larvae and attach them to the anatomical plate, and then separate its hemolymph, midgut and fat body. To analyze its expression profile during its different development stages, we firstly adjust the larvae growth period to a consistent state, the Asian corn borers were sampled during egg stage, first to fifth larval stages, pupa and adult stages, respectively. The tissue of three worms were treated as a sample, and three biological replicates were performed for each treatment. The samples were immediately frozen in liquid nitrogen and stored at -80°C until RNA extraction.

### RNA Isolation and cDNA Synthesis

Total RNA was extracted using TRIzol^®^ reagent (Invitrogen) according to the manufacturer’s instructions, and then treated with RNase-free DNase I for 30min at 37°C (New England BioLabs, Beverly, MA, USA) to remove residual DNA.

First-strand cDNA was synthesized using Oligo(dT)18 primer and reverse transcriptase (Invitrogen). Before cDNA synthesis, 5 μg total RNA was treated with RQ1 RNase-free DNase (Promega), according to the manufacturer’s instructions, to ensure no DNA contamination. cDNA synthesis was then carried out with the purified RNA using the SuperScript III First-Strand Synthesis System (Invitrogen), following the manufacturer’s instructions. The RT reaction was performed using Mastercycler Gradient (Eppendorf).

### Real-Time Quantitative PCR

qRT-PCR assay for multiple genes were performed with the SYBR^®^ Premix Ex Taq™ II (Takara). To ensure the qRT-PCR quality, two or three primer pairs were designed for all of the amplification segments, but only one pair was used in the final test. All of the primer sequences for qRT-PCR are listed in **Supplementary Table S1**. Melting-curve analyses were performed for all of the primers. To normalize Ct values obtained for each gene, 18S rRNA expression levels were used. qRT-qPCR was carried out using Mastercycler^®^ ep realplex (Eppendorf). All qRT-PCR assays were repeated three times to eliminate mechanical errors. qRT-PCR reactions and data were analyzed according to the methods of Livak & Schmittgen (20) and Bustin et al. (21). The data were analyzed using a one-way analysis of variance (ANOVA) to assess treatment effects compared with the untreated control.

### Multiple Sequence Alignment and Phylogenetic Analysis

Multiple sequence alignment of 20 trypsin inhibitor-like cysteine rich domain proteins (TIL-type) from 12 species (**Supplementary Table S2**). The multiple sequence alignment is limited to regions surrounding the lepidopteran exon boundaries by Cluxtal X (22).

Phylogenetic analysis was performed using the tBlast-N algorithm to search all published NCBI databases, using the *O. furnacalis* ACB-TIL amino acid sequence as a query, 20 amino acid sequences from 12 insect species were selected for multiple sequence alignment analysis. The 21 selected sequences were aligned by the *MUSCLE* alignment software, phylogenetic analysis was performed using MEGA version 5.2, a Neighbor-joining tree was constructed using the Poisson model and tested by the bootstrap method, with 1000 replications (all sequences information are listed in **Supplementary Table S2**). All gaps were treated as missing data.

### dsRNA Preparation

dsRNAs were synthesized using the MEGAscript^®^ RNAi Kit (Ambion, Huntingdon, UK) according to the manufacturer’s instruction. T7 promoter sequences were tailed to each 5’ end of the DNA templates by PCR amplifications. Double-stranded enhanced green fluorescent protein (dsEGFP) was generated using pPigbacA3EGFP as the template. All the primer sequences are listed in **Supplementary Table S1**. Template DNA and single-strand RNA were removed from the transcription reaction by DNase and RNase treatments, respectively. dsRNA was purified using MEGA clear columns (Ambion, Austin, USA) and eluted in nuclease free water. dsRNA concentrations were measured using a Biophotometer (Eppendorf, Hamburg, Germany).

### Immune Challenge of Asian Corn Borer

The fifth-instar larvae of Asian corn borer were kept in starvation for 1 hour, and then put on ice for 20 minutes. After surface disinfection with 75% alcohol, each larva was injected with 2.0 µL heat inactivated bacteria of *Escherichia coli* (trans1-T1 strain) (OD_600_ = 1.2) (23), or fungal spore of *Metarrhizium anisopliae* (1.0 × 10^6^ spore/mL) (24), or *Bombyx mori* nucleopolyhedrovirus (BmNPV) (1.01 × 10^7^ plaque forming units) (25), or dsEGFP (3µg/µl) (26), respectively. 2.0 µL phosphate buffered saline (PBS) (pH 7.0) was injected as control. Three insects were collected as one treatment, each treatment was repeated three times. Each of the above solutions was injected into the posterior abdominal segment using a capillary needle. Fat body was collected and frozen in liquid nitrogen 6 h and 12 h after injection.

### Expression and Purification of Recombinant ACB-TIL

The recombinant ACB-TIL protein was produced in an *E. coli* expression system. At first, the signal peptide sequence was cleaved off, and two expression plasmids were constructed, pET30a-ACB-TIL and pET32a-ACB-TIL. Then the recombinant plasmids were transformed into *E. coli* BL21(DE3) cells, and induced by 0.5 mmol/L IPTG, respectively. After 12 h incubation at 16°C, cultures were harvested by centrifugation at 12,000g at 4°C for 5 min. Cell pellet (1.0 g) was resuspended in 10 ml sonication buffer (50 mM Tris–HCl, 500 mM NaCl, pH 7.0) and lysed by sonication on ice at 200 W for 12 min (sonication for 6 s and intermission for 6 s). After sonication, the supernatants were recovered by centrifugation at 12,000g at 4°C for 30 min to remove the insoluble fraction. The supernatant of the total cell extract was used for purification.

Purification was performed essentially according to the instruction of HisTrap HP column. A 10 ml column was filled with Ni Sepharose medium and equilibrated with binding buffer (20 mM Na_3_PO4, 500 mM NaCl, 5 mM imidazole, pH 7.4). The sample was applied to the pre-equilibrated column, washed with binding buffer, and the recombinant fusion protein was eluted by a linear gradient of 5-500 mM imidazole in buffer (20 mM Na_3_PO4, 500 mM NaCl, pH 7.4) at 1 ml/min. Both the flow-through and the eluted fractions were collected and analyzed by SDS–PAGE.

### Hemolymph Melanization and Inhibiting Effect Assay *In vitro*

To collect hemolymph, the fifth-instar larvae of untreated Asian corn borer were starving for 1 hour in a petri dish, and then put on ice for 20 minutes. After surface disinfection with 75% alcohol, hemolymph was collected from between the second and third abdominal sternites using a microsyringe. Hemolymph was centrifuged at 4°C 6,000g for 5 min, and the supernatant was diluted 5 times with sterile PBS and used for subsequent tests. 2 μL of the diluted supernatant was added into a chilled Eppendorftube containing 8 μl of phosphate buffered saline (PBS), BSA, glycerin, PTU, and ACB-TIL, respectively. Gently mix and keep in room temperature, 20 hours later, observe the degree of melanization.

### The *In Vivo* Activity of Purified ACB-TIL

To test the activity of ACB-TIL *in vivo*, 2 µL (0.6 mg/mL) purified recombinant ACB-TIL protein was injected into a fifth-instar larva of Asian corn borer, using ddH_2_O and BSA as control. One hour later, 2 µL of heat-inactivated *E. coli* cell (OD_600_ = 1.2) were injected into each of the above three treated insects (ACB-TIL, BSA, and ddH_2_O treated groups). Twenty hours after treatments, the hemolymph and fat bodies of treated insects were extracted for the following experiments.

Hemolymph was used for bacteriostatic assay. 2 µL hemolymph from the above three experiments were dropped onto the LB plate coated with *E. coli* cell (Trans1-T1 strain), and then incubated at 37°C overnight. Fat body was used to examine the expression levels of four antimicrobial peptide genes of *cecropin*, *attacin*, *gloverin* and *lebocin*.

## Results

### Expression Profiles of Asian Corn Borer Serine Protease and Protease Inhibitor

**Figure 1:**
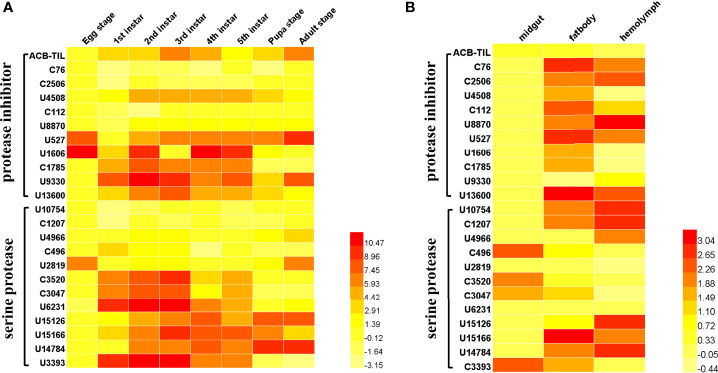
Spatiotemporal expression profile of serine protease and inhibitor genes in Asian corn borer. Heatmap analysis of gene expression of 11 protease inhibitors and 12 protease genes in different developmental stages **(A)** and different tissues **(B)** of Asian core borer. The color gradients indicate the gene expression levels. A darker red color represents higher gene expression, while a lighter yellow color represents lower gene expression. The Heatmap was generated by HemI 1.0 software, The value of the legend on the right side of the figure is calculated according to the qRT-PCR result, and the calculation formula is Log (2^−ΔΔCT^).

Through gene annotation and sequence alignment analysis of Asian corn borer transcriptome data (unpublished data), we obtained 12 protease genes and 11 protease inhibitor genes (**Supplementary Table S3**) (27). The expression profiles of all these genes in different development stages and in different tissues show in **Figure 1**. Some serine protease and protease inhibitor genes are highly expressed in the larval stage, like ACT-TIL, U4508, C1785, U9330, U13600, C3520, C3047, U6231, U15126, U15166, U14784, and U3393, etc. In addition, we also found that some genes were mainly expressed in the egg and adult stages, like U2819. From the tissue expression of those genes, most of these protease inhibitor genes were mainly expressed in the fat body and hemolymph, but some serine protease genes (like C496, C3520, C3047, C3393) are expressed in the midgut (**Figure 1**). In which, a previous unstudied serine protease inhibitor gene attracted our attention. It belongs to the canonical serine proteinase inhibitor family, a TIL-type protease inhibitors, named as *ACB-TIL*. Its expression level is relatively very low in different development stage and different tissues (**Figures 1A, B**).

### Inducible Expression of ACB-TIL by Different Immune-Related Factors

**Figure 2:**
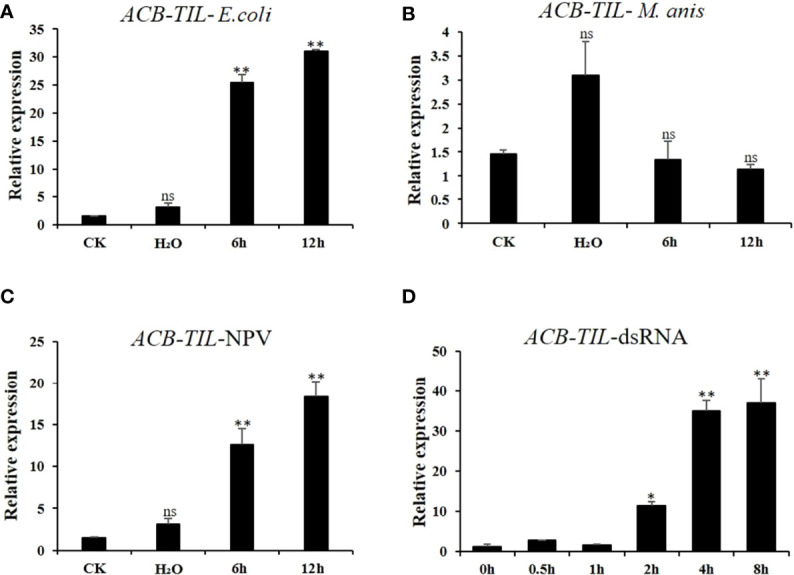
Effect of four different immune-related factor treatments on ACB-TIL gene expression in Asian corn borer. The expression level of *ACB-TIL* gene after bacteria (*Escherichia coli*) **(A)**, fungus (*Metarrhizium anisopliae* spores) **(B)**, virus (*Bombyx mori* NPV) **(C)** and dsRNA (dsEGFP) **(D)** treatments, respectively. CK: Negative control without any treatment. Data are Mean + SD, n=3, “*” indicates significant difference (P<0.05), “**” indicates extremely significant difference (P<0.01), “ns” indicates no significant difference. “6h, 12h” indicate 6h or 12h after bacteria, fungus or NPV treatments.

To clarify the relationship of *ACB-TIL* with insect immune response, the fifth instar larvae of Asian corn borer were challenged with inactive bacterial cell of *E. coli*, fungal spore of *M. robertisii*, nuclear polyhedrosis virus (BmNPV), and dsRNA (dsEGFP). The results indicated that the expression level of *ACB-TIL* can be induced by bacteria, virus and dsRNA, but cannot be induced by fungal spore (**Figure 2**). These results imply that ACB-TIL is just involved in the immune response of Asian corn borer to bacteria and virus.

### ACB-TIL Sequence Analysis and Protein Expression

**Figure 3:**
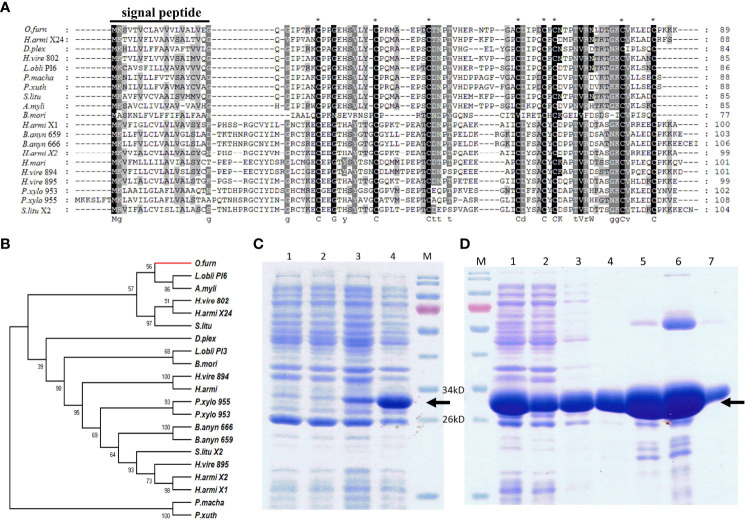
Sequence analysis and protein recombinant expression of ACB-TIL. Multiple sequence alignment **(A)** and Phylogenetic tree constructed **(B)** of TIL-type genes. SDS-PAGE analysis **(C)** and solubility assay of recombinant expressed ACB-TIL **(D)**. Black solid line indicates the signal peptides region; Black dot line indicates the TIL domain; “*” indicates the conserved cysteine residues. Lane 1 & 2 in **(C)**, 20 µL supernatant of *E*. *coli* cell lysate transfected by pET30a/ACB-TIL plasmid or pET30a/ACB-TIL (+) induced by 0.5 mmol/L IPTG; Lane 3 & 4 in **(C)**, 20 µL supernatant of *E*. *coli* cell lysate transfected by pET32a/ACB-TIL or pET32a/ACB-TIL (+) induced by 0.5mmol/L IPTG. Lane 1 in **(D)**, 20 µL supernatant of *E*. *coli* cell lysate transfected by pET32a/ACB-TIL (+) and induced by 0.5mmol/L IPTG. Lane 2 in **(D)**, the effluent from the column before eluted; Lane 3~7 in D, eluted solution by10, 20,50, 200 and 500 mM imidazole buffer, respectively. Lane M, protein molecular marker.

The full length 270 nucleotide cDNA sequence of *ACB-TIL* was obtained, it encodes a protease inhibitor containing 89 amino acids (GeneBank Accession No. MK411587). The first 1 to 19 amino acids were predicted as signal peptide by sequence analysis using SignalP 4.1 Server software (**Figure 3A**). Amino acids sequence analysis using Pfam software revealed that it contains a conserved TIL domain. The sequence identity of ACB-TIL with other protein with TIL domain (**Supplementary Table S2**) was analyzed. The result indicated that the sequence similarity is as high as 75% between ACB-TIL and a protease inhibitor 6 from *Lonomia oblique*. Usually, a TIL domain has ten conserved cysteine residues, and form five intradomain disulfide bridges, however, ACB-TIL lost two cysteine residues. A phylogenetic tree was constructed using 21 TIL domain SPIs from 12 species by the neighbor-joining method. The results indicated that ACB-TIL was gathered together in one branch with other five TIL proteins from *Lonomia obliqua*, *Antheraea mylitta*, *Heliothis virescens*, *Helicoverpa armigera*, and *Spodoptera litura* (**Figure 3B**; **Supplementary Table S2**).

To study the protease inhibitory activity of ACB-TIL, the ACB-TIL protein was expressed using a prokaryotic expression system. ACB-TIL is predicated as a 9.5 kD protein, the fusion protein with Trx-Taq, S-Taq and His-tag is about 30kD (**Figure 3C**, Lane 4). The fusion protein was soluble and can be eluted in large quantities by 50-200 mM imidazole buffer (**Figure 3D**, Lane 5-6).

### The Function of Recombinant ACB-TIL Protein *In Vitro* and *In Vivo*

**Figure 4:**
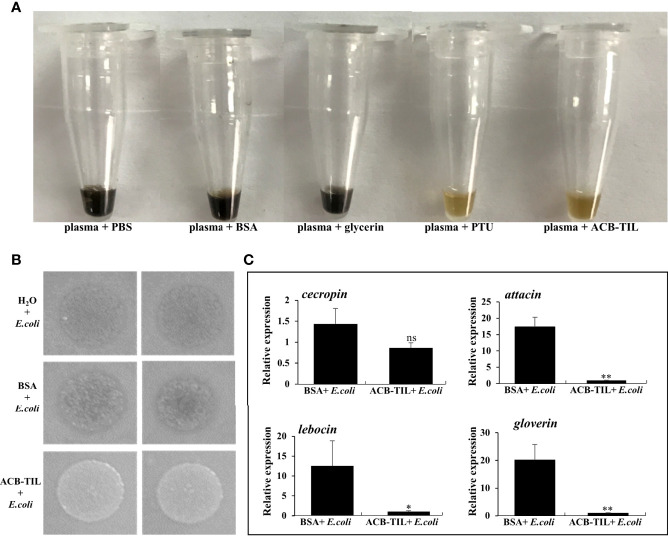
Function of recombinant ACB-TIL protein *in vitro* and *in vivo*. Effect of ACB-TIL protein on melanization assay **(A)**, bacteriostatic assay **(B)**, and the expression levels of four antimicrobial peptide genes in Asian corn borer **(C)**. Data are Mean + SD, n=3, “*” indicates significant difference (P<0.05), “**” indicates extremely significant difference(P<0.01), “ns” indicates no significant difference.

To verify the function of ACB-TIL, 2 μL purified recombinant protein was added into 8 μL Asian corn borer plasma PBS buffer, using PBS, BSA and glycerin as negative control, PTU as positive control, and then incubated at room temperature for 20 hours. The results showed that recombinant ACB-TIL protein can significantly inhibit the melanization of Asian corn borer hemolymph *in vitro* (**Figure 4A**).

We also tested the function of ACB-TIL *in vivo* through injected the purified recombinant protein into the insect body of fifth instar larvae of Asian corn borer. At first, 2 µL (0.6 mg/mL) purified ACB-TIL was injected into one insect body, using ddH_2_O and BSA as control. One hour later, 2 µL of heat inactive of *E. coli* cell (OD_600_ = 1.2) was injected into the insect body of the three different treatments, respectively. Twenty hours after treatment, the hemolymph and fat body were extracted for the following experiments.

Firstly, 2 µL hemolymph from above three treatments were dropped onto the LB plate coated with *E. coli* cell (Trans1-T1 strain), and then incubated at 37°C over night. Twelve hours later, obvious bacteria growth difference were observed, the bacteria grew more well on the LB plate dropped with hemolymph of ACB-TIL + *E.coli* treatment than on the other two control treatments (**Figure 4B**). So we deduced that the production of antimicrobial peptides in the insect body maybe inhibited by ACB-TIL protein, and leading to the decrease of anti-bacteria activity of the hemolymph.

Secondly, we examined the expression levels of four antimicrobial peptide genes in Asian corn borer fat bodies under the two treatments, ACB-TIL + *E. coli* and BSA + *E. coli*. Comparing with the treatment of BSA + *E. coli*, the expression level of cecropin is not impacted significantly by ACB-TIL + *E. coli*, however, three antimicrobial peptides gene of attacin, lebocin and gloverin are inhibited significantly by the treatment of ACB-TIL + *E. coli* (**Figure 4C**). These results indicated that ACB-TIL probably involved in antimicrobial peptide synthesis pathway, and inhibit its synthesis.

### Inhibition of ACB-TIL can Cause Responses of Other Immune-Related Genes

**Figure 5:**
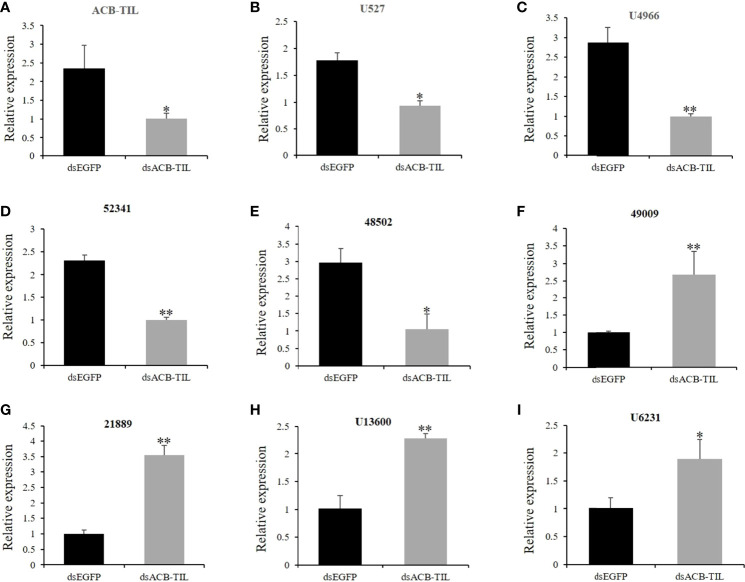
Relative expression levels of immune- or protease- or inhibitor-related genes after dsACB-TIL treatment. **(A-I)**, The gene annotations and predicted function information list in **Supplementary Tables S3, S4**. Data are Mean + SD, n=3, “*” indicates significant difference(P<0.05), “**” indicates extremely significant difference(P<0.01).

To further confirm the relationship of *ACB-TIL* with insect immune response, we synthesized some dsRNAs of *ACB-TIL*, 3 µL of 3µg/µl dsACB-TIL was injected into one fifth instar larvae of Asian corn borer. Post 4 hours of dsACB-TIL injection, the expression level of *ACB-TIL* was suppressed significantly (**Figure 5A**). At the same time, we also checked the expression levels of other eight insect immune- or protein inhibitor-related genes. Four of them are also suppressed, they are 48502 (E3 ubiquitin-protein ligase sina-like isoform X2), 52341 (E3 ubiquitin-protein ligase Siah1-like), U527 (trypsin inhibitor precursor), and U4966 (tryptase precursor); Four of them are up-regulated, they are 21889 (Gloverin-like protein), 49009 (peptidoglycan recognition protein B), U13600 (fungal protease inhibitor-1-like), and U4966 (putative trypsin 11) (**Figures 5B–I**; **Supplementary Table S4**).

## Discussion

In the present study, to discover and elucidate the application value of Asian corn borer endogenous protease and its inhibitor, the transcriptome sequences of Asian corn borer were analyzed in detail. We obtained 12 protease and 11 protease inhibitor genes of Asian corn borer and analyzed their spatiotemporal expression patterns (**Figure 1**). Most of these serine proteases and inhibitors were expressed in the fat body and hemolymph. We also found four serin proteases highly expressed in the midgut, and these proteins, have high expression levels in the larval stages (C3520, C3047, C3393). In terms of the developmental period, most of these proteases highly expressed in the larval stages, and some of them were able to sustain high expression into the adult stage. Three proteases (U527, U1606 and U2819) were expressed at high levels in the egg stage. However, there is one protease (U2819) that has a high expression only in egg and adult stages. The expression profiles of serine proteases and serine protease inhibitors have been studied in many insects, such as *Nilaparvata lugens* (28), *Plutella xylostella* (29), *Pteromalus puparum* (30), *Drosophila melanogaster* (31). In our studies, serin proteases and inhibitors display different tissue, and development-specific expression pattern, suggesting that these genes are widely involved in immune, developmental, and other physiological processes in Ascian corn borer. Our analysis of gene expression patterns of those genes can provide help for further study the gene function.

Among these protease inhibitors, *ACB-TIL* was cloned and further studied. The expression level of the gene can be up-regulated by bacteria, virus, and dsRNA, but not by fungal spore. dsRNA, like NPV, consists of nucleic acids that are capable of eliciting similar immune responses (32). In addition, the RNAi pathway induced by dsRNA is also an antiviral mechanism (33). In our results, both NPV and dsEGFP can induced *ACT-TIL* gene expression, and the same effect was observed in *E. coli*, suggesting that *ACT-TIL* may be closely related to the antiviral and antibacterial immune pathway in insects.

Using a prokaryotic protein expression system, the recombinant protein ACT-TIL was expressed and purified. The activity of purified protein was tested *in vitro* and *in vivo*, we confirmed that as the protease inhibitor, *ACB-TIL* can significantly inhibit the melanization *in vitro* (**Figure 4A**), and can inhibit the production of some antimicrobial peptides *in vivo* (**Figure 4C**), indicating *ACB-TIL* is involved in insect innate immune responses.

In insects, protease cascade bears the amplification and transmission of immune signals, and SPIs maintain normal immune response by regulating proteases activity to control the intensity of immune signals within a reasonable range (34). SPIs can able to negatively regulate the Toll pathway by inhibiting serine proteases activity, thereby affecting the production of antimicrobial peptides (34, 35). The prophenoloxidase (proPO) activation pathway is also regulated by SPIs. ProPO can be activated by specifically serine proteases into active PO, which in turn to catalyze melanization to defense against foreign invading pathogens (36–39). Many serpins have been shown to regulate the proPO cascade (40–43), but less studies focus on the function of small molecular weight SPIs containing a conserved TIL domain.

In our results, *ACB-TIL*, a TIL domain protease inhibitor was studied. In general, the TIL domain contain ten conserved cysteine residues and is capable of forming five intradomain disulfide bonds (they are 1-7, 2-6, 3-5, 4-10, and 8-9), and demonstrate inhibitory activities against cathepsin, trypsin, chymotrypsin or elastase (12, 44–46). In this study, the ACB-TIL lacked two cysteine residues (**Figure 3A**), which is similar to the two TIL domain protein BmSPI38 and BmSPI39 in *Bombyx mori*. In the proteins of BmSPI38 and BmSPI39, Cys^2nd^ and Cys^6th^ are replaced by other amino acids, functional validation of these two proteins revealed that they have the activities against microbial proteases and the germination of *Beauveria bassiana* conidia (12). From our results, and the ACB-TIL protein has the activity of inhibiting melanization *in vitro* and inhibiting antimicrobial peptides *in vivo* (**Figures 4A–C**). These results indicated that ACB-TIL may be involved in the melanization process and affect the expression of insect antimicrobial peptides in insects.

In our study, the spatiotemporal expression of serine proteases and inhibitors were analyzed, and the involvement of ACB-TIL in immune responses to Gram-negative bacteria and viruses of Asian corn borer was demonstrated. As a preliminary result, we hope that this study will provide some help for the research and application of endogenous proteases and inhibitors in Asian corn borer.

## Data Availability Statement

The original contributions presented in the study are included in the article/**Supplementary Material**. Further inquiries can be directed to the corresponding authors.

## Author Contributions

RG: Conceptualization, Formal analysis, Data Curation, Writing - Original Draft, Writing - Review and Editing. SH: Investigation, Formal analysis, Data Curation. HL: Formal analysis, Investigation, Writing - Original Draft. XL: Validation, Project administration. SA: Writing - Original Draft, Writing - Review and Editing. XM: Resources, Supervision. All authors contributed to the article and approved the submitted version.

## Funding

This work was supported by Shanghai Agriculture Applied Technology Development Program, China (Grant No. X2022-02-08-00-12-F01125), the Educational Commission of Henan Province of China (21A210017), the Science and Technology Planning Project of Henan Province of China (212102110442) and the National Natural Science Foundation of China (31772520; 31672354). The funders had no role in study design, data collection and analysis, decision to publish, or preparation of the manuscript.

## Conflict of Interest

The authors declare that the research was conducted in the absence of any commercial or financial relationships that could be construed as a potential conflict of interest.

## Publisher’s Note

All claims expressed in this article are solely those of the authors and do not necessarily represent those of their affiliated organizations, or those of the publisher, the editors and the reviewers. Any product that may be evaluated in this article, or claim that may be made by its manufacturer, is not guaranteed or endorsed by the publisher.
