# Supplementary material for: A TIL-Type Serine Protease Inhibitor Involved in Humoral Immune Response of Asian Corn Borer Ostrinia furnaculis
Source: Front Immunol. 2022 May 16;13:900129. doi: 10.3389/fimmu.2022.900129 (PMC9149172; doi:10.3389/fimmu.2022.900129)
Supplement: Supplementary file 1 [file DataSheet_1.docx]

**Supplementary Table S1. All primers used in this study***

|  | Gene Name | Forward Primer | Reverse Primer |
| --- | --- | --- | --- |
| 1 | ACB-TIL | ATGAAGTCTGTAACGGTGTG | TTACTTTTTCTTTGGACAGTC |
| 2 | C76 | GGTGGGCGACTCACACGACG | GCCTTTGGCGGAGCGAAGGT |
| 3 | C2506 | TCAACACGCTGTCCAAGTGCGA | GGAGCTCCGCCCCGAAATGG |
| 4 | U4508 | GCCATCGTGACTGTTCGCGGT | CCCTTGTGACCCCGTCGCAG |
| 5 | C112 | TTCCTCGACAGGGGAGCCCG | CGTTGGACGTGGCGGTCGAA |
| 6 | U8870 | GCCGGGGTTGGGGTACTGGA | TCCAACCGGGAGCACCACCA |
| 7 | U527 | TGCCGAAACAATTGCCGCTTTCA | TCGGAGGAGTCACTCGGGCA |
| 8 | U1606 | GCCCCAGATTGCACCTCGCC | ACGGTTGCCGTTGCCTTCACA |
| 9 | C1785 | TGACAACGCGGGAAAGGCGA | GGAGTGTCAGGCTGCTTGCGA |
| 10 | U9330 | ACGACGTGTACGCTGCTGCC | CGAGCAGCTGCCAAGCTGGA |
| 11 | U13600 | ATGGTTGGCGCGGTACACGG | CTGGCGTGTGTGGTGGCGAT |
| 12 | U10754 | GGCGGTGAGCACGTACCTCG | ATCGTGGGAGGGACCAGGGC |
| 13 | C1207 | TGCGCTGGTGGTGAAGCTGG | AGCCGATGCCCCAAGCAACC |
| 14 | U4966 | ATCGCTTCGTGCAAGCCGGT | ACGGCGGCCATCTTGTCGTC |
| 15 | C496 | GACACGCGCACATTGACGCC | TCTGGCGGGCCAGTCTTCCA |
| 16 | U2819 | CGCCCGATACCCACTTCGCC | TACTCCGCCTGCAGCTCCGT |
| 17 | C3520 | CAGCAACGAGGAGCCGCACA | CGCTGGACTTCGACGGCAGG |
| 18 | C3047 | TTCAGCAGCACCATCGCCCC | ACGGTGAGTCCGAAGCCCGA |
| 19 | U6231 | GCGCGGTACAGCGAGCTCTT | CAGAGGGCCGCCTTCGTCCT |
| 20 | U15126 | GTGGTCACTGCTCCCGCCAC | GACGGTCCACCACGGGCAAG |
| 21 | U15166 | ACACTCCGTATGGCCGGGGG | ACGGCGACAGTGGAGGTGGA |
| 22 | U14784 | AGAGACGCCTGCCTACGCGA | CCGCAGTTGTTGCCCCACGA |
| 23 | U3393 | GGTGTGACCTCCTTCGGTCTTGC | ACAGGTGCTCGTTGAAGAAGGGC |
| 24 | 18S | TCCCTGGTGTGATCCTGCCAGT | CTTCGAACGCGAGGCCCTCCGTC |
| 25 | Attacin | TCTCCATGGGCGTGAACTCG | CGATCTGTCCGATTGCGCTG |
| 26 | Cecropin | CGCTTGTTTCATGGCGTTCG | ACGATGCCGTCTCGGATGTT |
| 27 | Gloverin | GGAGACCTCACTGCTGACCA | GTGCTCGTAGCCAGCTTTGC |
| 28 | Lebocin | GATCCTGGCATTTGTGGCCG | GTTCGCTTCTTCGCACTCGG |
| 29 | 52341 | ATCTGCTCCGCCAGCTCCCA | GGGCCACCACAGATGCCACA |
| 30 | 48502 | GGCGGGCACAGGTTGTTGGT | CCGCAGGCCAAGTGGTGTCA |
| 31 | 49009 | CGGTGGGCGGAGATGGCAAG | CGCGGTGGCCGATGAGAGTG |
| 32 | 21889 | GCAGATCCGTTAGCCCGCCG | TCGTCAGACACACGAGGGACTTG |
| 33 | ds ACB-TIL | TAATACGACTCACTATAGGGAGAATGAAGTCTGTAACGG | TAATACGACTCACTATAGGGAGATTACTTTTTCTTTGGACAG |
| 34 | dsEGFP | TAATACGACTCACTATAGGGAGAAGCCGCTACCCCGACCACAT | TAATACGACTCACTATAGGGAGAGCCCCAGGATGTTGCCGTCC |
| 35 | ACB-TIL-30a/32a | GGGATCCCAAGGAATACCGACAAAAAAATG | CCGCTCGAGTTACTTTTTCTTTGGACAG |

*Primers 1-32 were used for quantitative detection; 33 and 34 were used for dsRNA synthesis, and 35 was used for ACB-TIL prokaryotic expression.

**Supplementary Table S2. *ACB-TIL* and other sequence information for multiple sequences alignment analysis.**

| Species | Abbreviation | Annotation | Sequence ID | Length(aa) |
| --- | --- | --- | --- | --- |
| *Ostrinia furnacalis* | O.furn | ACB-TIL | MK411587 | 89 |
| *Helicoverpa armigera* | H. arm B5X24 | hypothetical protein B5X24_HaOG202191 | PZC78447.1 | 88 |
| *Danaus plexippus plexippus* | D. plex | protease inhibitor protein | OWR52979.1 | 84 |
| *Heliothis virescens* | H. vire 8802 | hypothetical protein B5V51_8802 | PCG65706.1 | 85 |
| *Lonomia obliqua* | L. obli PI6 | protease inhibitor 6 | AAV91453.1 | 86 |
| *Papilio machaon* | P. macha | hypothetical protein RR48_13929 | KPJ17073.1 | 88 |
| *Papilio xuthus* | P. xuth | hypothetical protein RR46_13374 | KPI92153.1 | 88 |
| *Spodoptera litura* | S. litu | uncharacterized protein LOC111350535 | XP_022817924.1 | 85 |
| *Antheraea mylitta* | A. myli | protease inhibitor-like protein | ABG72723.1 | 85 |
| *Bombyx mori* | B. mori | fungal protease inhibitor F | NP_001037532.1 | 77 |
| *Helicoverpa armigera* | H. arm X1 | uncharacterized protein LOC110381998 isoform X1 | XP_021198121.1 | 100 |
| *Bicyclus anynana* | B. anyn9659 | uncharacterized protein LOC112049659 | XP_023943406.1 | 103 |
| *Bicyclus anynana* | B. anyn9666 | uncharacterized protein LOC112049666 | XP_023943419.1 | 106 |
| *Helicoverpa armigera* | H. arm X2 | uncharacterized protein LOC110381998 isoform X2 | XP_021198122.1 | 99 |
| *Helicoverpa armigera* | H. arm | uncharacterized protein LOC110381997 | XP_021198120.1 | 101 |
| *Heliothis virescens* | H. vire 6894 | hypothetical protein B5V51_6894 | PCG77592.1 | 101 |
| *Heliothis virescens* | H. vire 6895 | hypothetical protein B5V51_6895 | PCG77593.1 | 99 |
| *Plutella xylostella* | P. xylo4953 | uncharacterized protein LOC105394953 | XP_011565174.1 | 102 |
| *Plutella xylostella* | P. xylo4955 | uncharacterized protein LOC105394955 | XP_011565176.1 | 108 |
| *Spodoptera litura* | S. litu X2 | uncharacterized protein LOC111348824 isoform X2 | XP_022815493.1 | 104 |
| *Lonomia obliqua* | L. obli PI3 | protease inhibitor 3 | AAV91425.1 | 398 |

**Supplementary Table S3.** **Annotation and conserved protein domain information of protease and protease inhibitor genes screened from the transcriptome data of Asian corn borer.**

| Transcript-id number | annotation | Conserved Protein Domain Family |
| --- | --- | --- |
| ACB-TIL | protease inhibitor 6 [*Lonomia obliqua*] | TIL domain |
| C76 | serine proteinase inhibitor 1 [*O. furnacalis*] | SERPIN Superfamily |
| C2506 | serine protease inhibitor 3 [*O. furnacalis*] | SERPIN Superfamily |
| U4508 | serine protease inhibitor 7 precursor [*B. mori*] | SERPIN Superfamily |
| C112 | inter-alpha-trypsin inhibitor heavy chain H4-like isoform X5 [*Spodoptera litura*] | Vault protein inter-alpha-trypsin domain/Von Willebrand factor type A (vWA) domain |
| U8870 | protease inhibitor 1 precursor [*B. mori*] | KAZAL_FS Superfamily |
| U527 | trypsin inhibitor precursor [*B. mori*] | Kunitz/Bovine pancreatic trypsin inhibitor domain |
| U1606 | trypsin inhibitor-like isoform X1 [*B. mori*] | KU Superfamily |
| C1785 | Kunitz-type protease inhibitor precursor [*Galleria mellonella*] | KU Superfamily |
| U9330 | PREDICTED: serine protease inhibitor | KAZAL_FS Superfamily |
| U13600 | PREDICTED: fungal protease inhibitor-1-like [*Papilio machaon*] | amfpi-1 |
| U10754 | PREDICTED: serine protease easter-like isoform X2[*Amyelois transitella*] | Tryp_SPc Superfamily |
| C1207 | serine proteinase-like protein 1 [*Helicoverpa armigera*] | Tryp_SPc Superfamily |
| U4966 | tryptase precursor [*Papilio polytes*] | Tryp_SPc Superfamily |
| C496 | putative trypsin 14, partial [*O. nubilalis* | Tryp_SPc Superfamily |
| U2819 | serine proteinase [*Samia ricini*] | Tryp_SPc Superfamily |
| C3520 | serine protease 13 [*Antheraea pernyi*] | Tryp_SPc Superfamily |
| C3047 | chymotrypsin-like serine protease [*O. nubilalis*] | Tryp_SPc Superfamily |
| U6231 | putative trypsin 11 [*O. nubilalis*] | Tryp_SPc Superfamily |
| U15126 | tryptase-like, partial [*Pieris rapae*] | Tryp_SPc Superfamily |
| U15166 | serine proteinase [*Samia ricini*] | Tryp_SPc Superfamily |
| U14784 | clip domain serine protease 11 precursor [*B. mori*] | Tryp_SPc Superfamily |
| U3393 | chymotrypsin-like serine protease [*O. nubilalis*] | No putative conserved domains have been detected |

**Supplementary Table S4. Annotation and conserved protein domain information of four immune related genes screened from the transcriptome data of Asian corn borer.**

| Transcript-id number | annotation | Conserved Protein Domain Family |
| --- | --- | --- |
| 52341 | E3 ubiquitin-protein ligase Siah1-like | Sina Superfamily |
| 48502 | E3 ubiquitin-protein ligase sina-like isoform X2 | Sina Superfamily |
| 49009 | peptidoglycan recognition protein B | PGRP Superfamily |
| 21889 | T9SS type B sorting domain-containing protein | Gloverin-like protein |
